# Attrition and Success Rates in the Saudi Board of Neurosurgery: Analysis of 115 Consecutive Residents Who Started Training From 2001 to 2014

**DOI:** 10.7759/cureus.18235

**Published:** 2021-09-24

**Authors:** Abdulhadi Y Algahtani, Abdulhakim B Jamjoom, Abdulkarim Al Rabie, Zain Alabedeen B Jamjoom

**Affiliations:** 1 Neurosurgery, King Saud Bin Abdulaziz University for Health Sciences College of Medicine, Jeddah, SAU; 2 Neurosurgery, Prince Sultan Military Medical City, Riyadh, SAU; 3 Neurosurgery, King Faisal Specialist Hospital and Research Centre, Jeddah, SAU

**Keywords:** success rates, attrition rates, neurosurgery certification, saudi arabia, saudi board in neurosurgery

## Abstract

Objectives

At present, the literature lacks data on the outcome of neurosurgery training programs in the Middle East. In this study we aim to assess the attrition, completion of training and success rates in the Saudi Board of Neurosurgery (SBNS).

Methods

A cohort of 115 trainees who started SBNS training during 2001-2014 was reviewed. The outcome was the rate of attrition, completion of training, and success in the final examination of the SBNS.

Results

Attrition rate was 29% (14% to neurosurgery training elsewhere and 15% to non-neurosurgery). Completion of training rate was 71%. Success in the final examination rate was 74% (60% on the first attempt). Attrition rate was significantly influenced by being sponsored by University Hospitals. Success rate was impacted positively by being sponsored by King Fahad Medical City and negatively by Ministry of Health Hospitals. Trainees who started during 2011-2014 had a significantly better success rate in the final examination.

Conclusions

SBNS attrition rate was high due to access to training opportunities abroad, particularly for university-sponsored trainees. Success rate in the final examination was considered comparable to some other neurosurgical qualifications. The first attempt pass rate was significantly impacted by being sponsored by certain hospitals. Factors contributing to attrition and failure should be identified and addressed during the selection process and during training.

## Introduction

The density of neurosurgeons in the Kingdom of Saudi Arabia (KSA) was calculated as 1 per 250,000 population [1]. The relative paucity of neurosurgeons had been attributed to the country’s reliance on the limited opportunities for neurosurgical training abroad. Increasing demands from a rapidly growing population necessitated the conception of a national training program in neurosurgery. The first neurosurgical program in KSA was a 1987 collaborative effort between King Faisal University, Dammam and King Faisal Specialist Hospital and Research Centre (KFSH & RC), Riyadh. This was later modified and launched as the Saudi Board of Neurosurgery (SBNS) in 1995.

The SBNS training program is a standardized curriculum that is set and administered by the Saudi Commission for Health Specialties (SCFHS) [2]. It is conducted at accredited institutions that are in major cities where several large hospitals can complement each other to fulfill the training requirements. Up until recently, accredited training hospitals were clustered into one of the three main regions in the country (central, western and eastern). The selection process for the SBNS training is centralized and overseen by the SCFHS. Candidates are initially nominated for training based on their university’s grade point average (GPA), Saudi Medical Licensing Examination (SMLE), and curriculum vitae (CV) scores. Shortlisted candidates then compete for one of the sponsored positions that are annually released by the various training hospitals. The main hospitals that participate in the SBNS training are Ministry of Health Hospitals (MOHH), King Fahad Medical City (KFMC), KFSH & RC, University Hospitals (UH), Prince Sultan Military Medical City (PSMMC), and King Abdulaziz Medical City (KAMC). The SBNS is a six-year structured training program, divided into a three-year junior and a three-year senior residency levels. Trainees must pass the Part 1 examination during the junior training, and it is mandatory for promotion to residency R4 level. Following the completion of residency, trainees must pass the final SBNS examination which consists of a written multiple-choice questions (MCQ) section and an Objective Structured Clinical Examination (OSCE) section. Passing the final examination is mandatory for the SBNS certification [2].

Rates of completion of training and success in the final examination are indicators of the quality of the selection process, the training program and the qualification standard [3,4]. At present, the literature lacks data on the outcome of neurosurgery training programs in the Middle East. The objective of this study was to determine the attrition, completion of training and success rates in the final SBNS examination and to ascertain whether the outcome was impacted by several trainee and training center characteristics.

## Materials and methods

The study was carried out during October- December 2020. Data relating to the completion of training and success in the final examination was compiled by the two senior authors (ABJ and ZBJ) who served in the SCFHS organizing committee for the SBNS during most of the study period. Demographic data of the cohort was obtained utilizing an online survey that was sent to all trainees that started the SBNS training program during 2001-2014. Trainees were identified by their training number and confidentiality was maintained throughout.

Attrition rate was defined as the proportion of trainees who dropped out of the SBNS training any time after starting and before completion of training. Trainees who left the SBNS and started training in another specialty or took clinical service posts were referred to as the attrition to the non-neurosurgery group. Trainees who moved out of the SBNS and continued their neurosurgery training in another country were referred to as the attrition to neurosurgery elsewhere group. The following data was collected: age at starting training, gender, nationality, medical college attended, year of graduation, GPA score, SMLE score, year of starting training, duration from graduation to starting training, training region, sponsoring hospital, attrition to neurosurgery elsewhere, attrition to non-neurosurgery, year of completion of training, year of passing the final SBNS examination, number of attempts, and passing of other neurosurgery qualifications.

The outcome was the rates of attrition, completion of training and passing of the final SBNS examination. The impact of several trainee and training center characteristics on the outcome was assessed by dividing the cohort into domains or groups based on the value of the median and comparing them statistically. The comparison was carried out using a chi-squared test from an online source [5]. In all the statistical analyses, significance was achieved at P < 0.05. 

## Results

The total number of trainees that started the SBNS training program during 2001-2014 was 115. Response rate to the survey was 86(75%). Complete accurate data relating to gender, nationality, year of starting training, training region, sponsoring hospital, attrition and success rate was available for the trainees in this study. The data that was influenced by the response rate and was not available for the whole cohort was limited to age, duration from graduation to starting training, GPA and SMLE scores. The median (range) age at the start of training was 25 (24-32) years. The median (range) GPA score was 80% (56%- 94%) for the whole cohort and 75% (56%- 90%) for MOHH-sponsored trainees. The median (range) SMLE score was 77% (53%- 91%). The medical colleges attended were King Saud University: 23 (20%), King Abdulaziz University: 22 (19%), Imam Abdulrahman bin Faisal University: 9 (7.8%), Umm AlQura University: 9 (7.8%), King Khalid University: 7 (6.1%), Taiba University: 6 (5.2%), King Saud bin Abdulaziz University for Health Sciences: 6 (5.2%), King Faisal University: 6 (5.2%), Taif University: 1 (0.9%), Qassim University: 1 (0.9%), Jouf University: 1 (0.9%), Ibn Seina University: 1 (0.9%), overseas universities: 8 (7%) and unknown: 15 (13%). The median (range) year of graduation was 2009 (2000- 2013). The median (range) year of starting training was 2011 (2001-2014). The median (range) duration from graduation to starting training was 1 (1-7) year. The median (range) annual number of trainees was 7 (2-21). Data related to gender, nationality, training region and sponsoring hospital are summarized in Table 1.

**Table 1:** Characteristics of the cohort of 115 residents who started the SBNS training during 2001- 2014. SBNS: Saudi Board of Neurosurgery

Feature	Parameters	Number (%)
Gender	Male	91(79%)
	Female	24(21%)
Nationality	Saudi	103(90%)
	Non-Saudi	12(10%)
Training Region	Central	70(61%)
	Western	27(24%)
	Eastern	18(16%)
Sponsoring Hospital	Ministry of Health Hospitals	32(28%)
	King Fahad Medical City	20 (17%)
	King Faisal Specialist Hospital and Research Centre	19(17%)
	University Hospitals	16(14%)
	Prince Sultan Military Medical City	14(12%)
	King Abdulaziz Medical City	7(6%)
	Unknown	7(6%)

The overall attrition rate was 33 (29%). The rate of attrition to neurosurgery elsewhere and to non-neurosurgery were 16 (14%) and 17 (15%) respectively. Table 2 shows the country and alternative career destinations for the trainees who attrited to neurosurgery elsewhere and to non-neurosurgery. The median (range) year of attrition was 2011 (2003-2014) and the median (range) residency level at the time of attrition was R2 (R1-R5).

**Table 2:** The country and alternative career destinations for the 33 SBNS trainees who attrited to neurosurgery elsewhere and to non-neurosurgery. SBNS: Saudi Board of Neurosurgery

Group	Total	Country/ Alternative Career Destination	Number
Attrition to neurosurgery elsewhere	16(14%)	Canada	14
		France	2
Attrition to non- neurosurgery	17(15%)	Primary Care	4
		Neurosurgery service post	2
		Anesthesia	1
		Emergency Medicine	1
		Neurology	1
		General Surgery	1
		Ophthalmology	1
		Orthopedics	1
		Preventive Medicine	1
		Non-Medical job	1
		Unknown	3

The overall rate of completion of SBNS training was 82 (71%). The median (range) duration of completion of training was 6 (6- 9) years. Of the 27 (33%) trainees that did not complete the training in 6 years, 23 (28%) completed it in 7 years, 2 (2%) in 8 years, and 2 (2%) in 9 years. The median (range) year of completion of training was 2017 (2007-2020). The impact of several trainee and training center characteristics on attrition and completion of training rates is summarized in Table 3. Trainees that were sponsored by University Hospitals compared to others had a statistically significantly higher rates of attrition to neurosurgery elsewhere (38% vs. 10%), and lower rates of attrition to non-neurosurgery (0% vs. 17%) (P=0.006). None of the other parameters reached significance.

**Table 3:** Impact of several trainee and training centre characteristics on the completion of training and attrition rates for 115 SBNS trainees. In numerical domains, the cohort was divided into two groups based on the value of the median. Abbreviations: N: Number of trainees with available data, GPA: grade point average, SMLE: Saudi Medical Licensing Examination, MOHH: Ministry of Health Hospitals, KFMC: King Fahad Medical City, KFSH & RC: King Faisal Specialist Hospital and Research Centre, UH: University Hospitals, PSMMC: Prince Sultan Medical City, KAMC: King Abdulaziz Medical City, X2: Chi-squared, SBNS: Saudi Board of Neurosurgery a: N=83, b: N=82, c: N=68, d: N=86; *: Significant

Feature	Domains	Total Number	Completed Training	Attrition to Neurosurgery Elsewhere	Attrition to Non-Neurosurgery	X2 P- value
Age^a^	<25 years	16	13(81%)	3(19%)	0(0%)	X2=1.97 P=0.374
	≥25 years	67	51(76%)	9(13%)	7(10%)	
Gender	Male	91	67(74%)	14(15%)	10(11%)	X2=5.25 P=0.072
	Female	24	15(63%)	2(8%)	7(29%)	
Nationality	Saudi	103	72(70%)	16(16%)	15(15%)	X2=2.17 P=0.338
	Non-Saudi	12	10(83%)	0(0%)	2(17%)	
GPA score^b^	< 80%	40	35(88%)	2(5%)	3(8%)	X2=5.85 P=0.054
	≥80%	42	29(69%)	10(24%)	3(7%)	
SMLE score^c^	< 77%	32	27(84%)	3(9%)	2(6%)	X2=1.46 P=0.483
	≥ 77%	36	26(72%)	6(17%)	4(11%)	
Year of starting training	2001- 10	56	41(73%)	9(16%)	6(11%)	X2=1.64 P=0.440
	2011- 14	59	41(70%)	7(12%)	11(19%)	
Duration from graduation to starting^d^	1 year	66	47(71%)	14(21%)	5(8%)	X2=3.27 P=0.195
	>1 year	20	18(90%)	2(10%)	0(0%)	
Training region	Central	70	52(74%)	8(11%)	10(14%)	X2=1.63 P=0.804
	Western	27	19(70%)	4(15%)	4(15%)	
	Eastern	18	11(61%)	4(22%)	3(17%)	
Sponsoring hospital	MOHH	32	25(78%)	2(6%)	5(16%)	X2=2.18 P=0.336
	Others	83	57(69%)	14(17%)	12(15%)	
	KFMC	20	13(65%)	2(10%)	5(25%)	X2=2.11 P=0.348
	Others	95	69(73%)	14(15%)	12(13%)	
	KFSH & RC	19	13(68%)	4(21%)	2(11%)	X2=1.16 P=0.559
	Others	96	70(73%)	12(13%)	15(16%)	
	UH	16	10(63%)	6(38%)	0(0%)	X2=10.38 P=0.006*
	Others	99	72(73%)	10(10%)	17(17%)	
	PSMMC	14	13(93%)	0(0%)	1(7%)	X2=3.89 P=0.143
	Others	101	69(68%)	16(16%)	16(16%)	
	KAMC	7	5(71%)	1(14%)	1(14%)	X2=0.002 P=0.999
	Others	108	77(71%)	15(14%)	16(15%)	

The overall success rate in the SBNS final examination after completion of training was 61(74%). The pass rates on the first attempt and after the first attempt were 49 (60%) and 12 (15%), respectively. Table 4 summarizes the success rates in the final examination for 82 residents who completed training. The rate of passing another neurosurgery qualification among trainees who completed the SBNS training was 15/82 (18%). The rate among those who attrited to neurosurgery elsewhere was 10/16 (63%). The overall rate of passing another neurosurgery qualification was 25/98 (26%), which was low because the SBNS is the standard neurosurgery certification in Saudi Arabia and getting another neurosurgery qualification is optional. The other neurosurgical qualifications in this cohort were European Board: 10 (10%), Canadian Fellow of the Royal College of Surgeons of Canada [FRCS(C)]: 8 (8%), Jordanian Board: 4 (4%), Arab Board: 3 (3%), UK’s Intercollegiate FRCS(SN): 2 (2%), French Board: 1 (1%) and King Faisal University (KFU) Fellowship: 1 (1%). Four (4%) trainees had more than one other qualification.

**Table 4:** Summary of success rates in the SBNS final examination for 82 residents who completed training. SBNS: Saudi Board of Neurosurgery

Feature	Total Number	Completed Training in 6 Years	Completed Training in > 6 Years
Completed SBNS Training Program	82	55(67%)	27(33%)
Passed SBNS final examination (all)	61	43(70%)	18(30%)
Passed SBNS final examination (first attempt)	49	36(73%)	13(27%)
Not passed SBNS final examination	21	12(57%)	9(43%)

The impact of several trainee and training center characteristics on the success rates in SBNS final examination for the 82 residents who completed the SBNS training during 2007-2020 is summarized in Table 5. Trainees starting during the period 2011-2014 compared to those that started during 2001-2010 had statistically significantly higher rates of passing the final examination on the first attempt (68% vs. 51%) (P=0.041). Furthermore, trainees sponsored by KFMC compared to others had statistically significantly higher rates of passing the final examination on the first attempt (92% vs. 54%) (P=0.027). Conversely, trainees sponsored by MOHH had statistically significantly higher rates of not passing the final examination (44% vs. 18%) (P=0.041). None of the other parameters reached significance.

**Table 5:** Impact of several trainee and training center characteristics on success rates in SBNS final examination for the 82 residents who completed the SBNS training. In numerical domains, the cohort was divided into two groups based on the value of the median. Abbreviations: N: Number of trainees with available data, GPA: grade point average, SMLE: Saudi Medical Licensing Examination, MOHH: Ministry of Health Hospitals, KFMC: King Fahad Medical City, KFSH: King Faisal Specialist Hospital and Research Centre, UH: University Hospitals, PSMMC: Prince Sultan Medical City, KAMC: King Abdulaziz Medical City, X2: Chi-squared, SBNS: Saudi Board of Neurosurgery a: N=64, b: N=64, c: N=53, d: N=65, *: Significant

Feature	Domains	Total Number	Passed SBNS on first attempt	Passed SBNS after first attempt	Not Passed SBNS	X2 P- value
Age^a^	<25 years	13	11(85%)	1(8%)	1(8%)	X2=2.32 P=0.314
	≥25 years	51	32(63%)	7(14%)	12(24%)	
Gender	Male	67	40(60%)	10(15%)	17(25%)	X2=0.03 P=0.985
	Female	15	9(60%)	2(13%)	4(27%)	
Nationality	Saudi	72	44(61%)	11(15%)	17(24%)	X2=1.27 P=0.53
	Non-Saudi	10	5(50%)	1(10%)	4(40%)	
GPA score^b^	< 80%	35	23(66%)	5(14%)	7(20%)	X2=0.365 P=0.833
	≥80%	29	21(72%)	3(10%)	5(17%)	
SMLE score^c^	< 77%	27	16(59%)	5(19%)	6(22%)	X2=0.69 P=0.708
	≥ 77%	26	18(69%)	3(12%)	5(19%)	
Year of starting training	2001- 10	41	21(51%)	10(24%)	10(24%)	X2=6.38 P=0.041*
	2011- 14	41	28(68%)	2(5%)	11(27%)	
Duration from graduation to starting^d^	1 year	47	35(75%)	4(9%)	8(17%)	X2=3.89 P=0.143
	>1 year	18	9(50%)	4(22%)	5(28%)	
Training region	Central	52	35(67%)	6(12%)	11(21%)	X2=0.48 P=0.479
	Western	19	9(47%)	4(21%)	6(32%)	
	Eastern	11	5(46%)	2(18%)	4(36%)	
Sponsoring hospitals	MOHH	25	11(44%)	3(12%)	11(44%)	X2=6.41 P=0.041*
	Others	57	38(67%)	9(16%)	10(18%)	
	KFMC	13	12(92%)	1(8%)	0(0%)	X2=7.20 P=0.027*
	Others	69	37(54%)	11(16%)	21(30%)	
	KFSH & RC	13	8(62%)	1(8%)	4(31%)	X2=0.68 P=0.713
	Others	69	41(59%)	11(16%)	17(24%)	
	UH	10	6(60%)	2(20%)	2(20%)	X2=0.36 P=0.834
	Others	72	43(60%)	10(14%)	19(26%)	
	PSMMC	13	10(77%)	2(15%)	1(8%)	X2=2.71 P=0.259
	Others	69	39(57%)	10(15%)	20(29%)	
	KAMC	5	2(40%)	2(40%)	1(20%)	X2=2.75 P=0.252
	Others	77	47(61%)	10(13%)	20(26%)	
Duration of training	6 Years	55	36(65%)	7(13%)	12(22%)	X2=2.26 P=0.323
	>6 Years	27	13(48%)	5(19%)	9(33%)	
Year of completion of training	2007- 16	35	20(57%)	8(23%)	7(20%)	X2=3.64 P=0.162
	2017- 20	47	29(62%)	4(9%)	14(30%)	
Other neurosurgery qualifications	Yes	15	9(60%)	1(7%)	5(33%)	X2=1.22 P=0.542
	No	67	40(60%)	11(16%)	16(24%)	

## Discussion

Neurosurgical training is demanding because of the critically ill patient population, the technically stressful operations and the high-volume workload. Yet it remains competitive and continues to attract motivated students who are committed to undergo intense training. There is a worldwide variation in the complexity and standards of neurosurgery certification [6-9]. Based on a survey of members of the World Federation of Neurosurgical Societies (WFNS), a global regional complexity score (RCS-G), ranging from 0 to 40, was calculated for neurosurgery examinations all over the world. The RCS-G was the sum of several scores in domains relating to the organization and components of the neurosurgery examination [6-9]. The RCS-G for KSA was 16 points [8]. In a global context and based on these studies, the SBNS qualification could be ranked in the mid-range of complexity compared to other worldwide neurosurgery examinations. It is almost certainly less complicated than the certification process in high scoring countries (their RCS-G score) such as: South Korea (21), Malaysia (21), Singapore-Hong Kong (20), USA (19), UK (19), Brazil (19), South Africa (19), Canada (18), Mexico (17) and Japan (17) [6-9]. However, it can be considered comparable in difficulty to that in countries such as Poland (16), Sweden (16), Portugal (15) and Philippines (15) [6,9], and possibly more challenging than the certification process in countries such as Switzerland (14), Taiwan (13), Nigeria (11), Turkey (9) and Germany (7) [6,8,9].

We observed a relatively high attrition rate to neurosurgery elsewhere (14%) that was significantly higher among University Hospitals sponsored trainees (38% vs. 10%). This can be attributed to the active government-sponsored scholarship program that gave distinguished graduates the opportunity to train abroad. Also, to the Saudi universities’ preference for their future faculty to acquire a more reputable international qualification. Attrition rate outside neurosurgery was 15% which is comparable to the 14% reported for neurosurgery residency in the USA during 1990-1999 [4], but higher than the 7% reported for the same during 2000-2009 [10]. The median residency level at the time of attrition in this series was R2 which is like the findings by others [10].

The overall pass rate in the SBNS final examination was 74% and the first attempt pass rate was 60%. This was lower than the Canadian FRCS(C) which was reported as 98% during 2017-2019 [11]. It was also lower than the American Board in Neurosurgery (ABNS) oral examination which was reported as 81%-85% during 2017-2019 [12]. The SBNS first attempt pass rate was, however, higher than the UK’s FRCS(SN) which was reported as 50% in 2017 [13]. The first attempt pass rate was significantly higher among KFMC trainees (92% vs. 54%) and significantly lower among MOHH trainees (44% vs. 67%) compared to others. These findings indicate that the performance of SBNS trainees was significantly impacted by being trained at a certain hospital. A similar variation in the FRCS(SN) first attempt pass rates was observed between different UK deaneries [13]. The SBNS program is centralized, nevertheless, trainees spend longer periods of training at their sponsoring hospital. The MOHH sponsored the largest number in this cohort (28%). The median GPA for MOHH trainees was slightly lower than that for the whole cohort (75% vs. 80%). It is, therefore, possible that the lower performance of its trainees may be related to the selection process. However, the variation could be a sign of the diverging standard of neurosurgical expertise provided by the various hospitals in KSA. This is not surprising as the regional and national unfair distribution of workforce and workload in neurosurgery is recognized worldwide [1]. The SCImago Institutions Ranking (SCR) ranked five KSA healthcare facilities among the top 40 institutions in the Middle East in 2020. These included a few of the SBNS training hospitals (KFSH & RC, KFMC, KAMC and King Khalid UH) [14].

The findings here need to be interpreted with caution as evaluating the performance of institutions based on one or two factors may be unfair [13], and the passing of SBNS on the first attempt may not necessarily correlate with a successful career or safe practice. Nevertheless, the highlighting of the performance of institutions in the SBNS examination provides a basis that demonstrates responsibility, achievements and quality assurance, and may contribute to the improved condition by stimulating inter-institutional competition [13]. It also offers a crucial factor for prospective candidates to weigh when choosing a training location [13]. We believe that the impact of the training hospitals on success rate in SBNS is something the program developers and trainers must address to avoid having a two-tier standard of graduates. A strict rotation system that allows trainees to spend equal periods of training at the major high-volume neurosurgical centers should be implemented. Furthermore, the trainees’ selection process should continue to be thorough, adapts to new demands and learn from the experience of other countries [3].

Women accounted for 21% of this cohort which is higher than the percentage of women (12%) selected for neurosurgery residency in the USA during 2000-2009 [10]. The rate of women leaving neurosurgery in this study was slightly higher than in the USA (29% vs. 24%) [4]. No significant difference in attrition and success rates between males and females was observed in this cohort. These findings differ from reports from USA which showed that women compared to men had significantly higher attrition rates (17% vs. 5%) [10], and a significantly lower ABNS certification rate (63% vs. 81%) [4].

In this study, the attrition and success rates were not impacted by the GPA or the SMLE scores. The findings may have been biased by the unavailability of all the scores for the whole cohort. The correlation between academic background and performance in the neurosurgery examination is a matter of debate. It has been reported that the United States Medical Licensing Examination (USMLE) Step 1 directly correlates with the ABNS scores [15], while the USMLE Step 2 was not considered a reliable predictor of performance in the ABNS written examination [16]. In addition, alumni of USA public medical school had significantly higher board certification rates compared to private and international (82% vs. 77% and 78% respectively) [4].

There was a statistically significantly higher first-attempt success rate among trainees who started SBNS training during 2011-2014 compared to those that started during 2001-2010 (68% vs. 51%). However, there was no difference in success rates between trainees who completed training in 6 years or more which agrees with findings by others [4]. The temporal changes in attrition and success rates are influenced by many factors such as competition ratio, selection policy, and the availability of overseas scholarship opportunities. However, it is possible that some of the superior success rates are attributed to progress in workload, workforce and quality of training in the SBNS program.

The study has several limitations. Being retrospective, some of the data relating to age, GPA, and SMLE scores was reliant on self-reporting surveys and were incomplete. During the relatively long study period, changes may have occurred in selection policy, training rotations, working conditions, and inclusion of new training hospitals which may have affected the outcome. The study included candidates that started training and the few candidates that were selected but did not start training were not included. The cohort is relatively small with a diverse background relating to their medical degree hence the impact of the medical college on success rates could not be assessed. Trainees who completed training in 2020 and did not pass on the first attempt were considered as did not pass. The outcome was based on passing the SBNS examination, particularly on the first attempt, which may not necessarily reflect the quality of training. The study did not include a follow-up on the career progress of the trainees after passing the SBNS final examination. Further studies that cover larger study populations are required to validate the findings of this study.

## Conclusions

This is the first report of the outcome of postgraduate medical specialty training in KSA. Attrition rate in the SBNS training program was relatively high due to access to training opportunities abroad, particularly to university-sponsored trainees. Of the 71% completed the SBNS training, 60% passed on first attempt and 26% had not passed at the time of conclusion of the study. The success rate could be considered comparable some of the other qualifications. First attempt pass rates were significantly influenced by being a trainee at certain hospitals and the timing of training. We recommend that SBNS trainees should get equal periods of training at the major high-volume neurosurgical centres. Factors contributing to attrition and failure to pass the examination should be identified and addressed in the selection process and during training.
